# ddpcr: an R package and web application for analysis of droplet digital PCR data

**DOI:** 10.12688/f1000research.9022.1

**Published:** 2016-06-17

**Authors:** Dean Attali, Roza Bidshahri, Charles Haynes, Jennifer Bryan

**Affiliations:** 1 Bioinformatics Training Program, University of British Columbia, Vancouver, Canada; 2 Michael Smith Laboratories, University of British Columbia, Vancouver, Canada; 3 Department of Chemical and Biological Engineering, University of British Columbia, Vancouver, Canada; 4 Department of Statistics, University of British Columbia, Vancouver, Canada

**Keywords:** droplet digital PCR, shiny, bioinformatics, personalized medicine, rpackage, gating, Gaussian mixture models, kernel density estimates

## Abstract

Droplet digital polymerase chain reaction (ddPCR) is a novel platform for exact quantification of DNA which holds great promise in clinical diagnostics. It is increasingly popular due to its digital nature, which provides more accurate quantification and higher sensitivity than traditional real-time PCR. However, clinical adoption has been slowed in part by the lack of software tools available for analyzing ddPCR data. Here, we present *ddpcr *– a new R package for ddPCR visualization and analysis. In addition, *ddpcr *includes a web application (powered by the Shiny R package) that allows users to analyze ddPCR data using an interactive graphical interface.

## Introduction

Droplet digital polymerase chain reaction (ddPCR) accurately quantifies targeted nucleic acid sequences (templates) by randomly partitioning sample DNA into isolated droplets, such that most droplets contain at most one template. The template within each droplet is then amplified and detected in a sequence-specific manner using a hydrolysis probe. The counting of droplets emitting a sequence-specific fluorescent signal permits the number of copies of that sequence present in the sample to be quantified with excellent sensitivity and precision. Different templates, such as wild-type and mutant alleles, may be quantified by using a uniquely labeled probe against each. The most commonly used reporter dyes on the probes are FAM (fluorescein) and HEX™, with the end-point fluorescence amplitudes for the two dyes measured by analyzing each droplet with a two-channel fluorescence detector ^1^.

**Figure 1.**
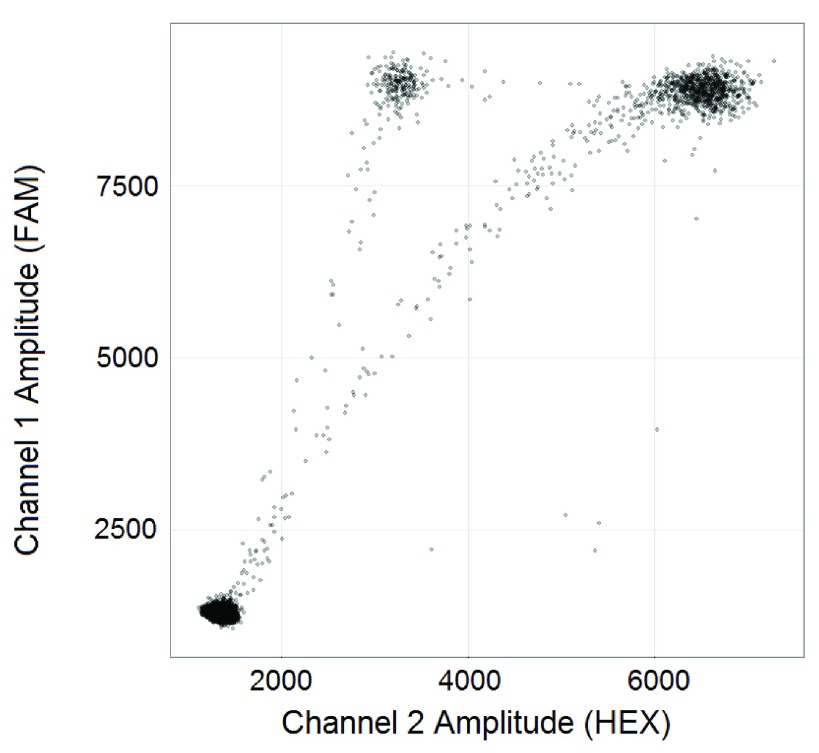
Raw ddPCR data from a two-channel ddPCR experiment (well F05 from the sample dataset).

ddPCR data readily lends itself to visualization as a two-dimensional scatter plot ( Figure 1), in which the fluorescence amplitudes in both channels are plotted against each other for every droplet. In a ddPCR experiment designed to quantify two different templates, droplets ideally segregate into unique groups (clusters) that may include HEX-positive, FAM-positive, double-positive, and double-negative (empty) clusters ^2^. For example, distinct FAM-positive, double-positive, and empty droplet clusters can be seen in Figure 5B. In practice, some droplets record an ambiguous set of fluorescent signals that fall between the distinct positive and negative populations. Such droplets are termed “rain” and can be observed between all clusters. By gating the droplets into groups based on their fluorescence signals, the exact number of template-positive droplets can be counted to provide exact quantification in a digital form.

## Motivation

Quantification of template abundance from raw ddPCR data begins with assigning each droplet to a unique cluster or to rain. The QuantaSoft program (Bio-Rad, Hercules, CA) is designed to perform these assignments either via manual gating, with the usual disadvantages of subjectivity and non-reproducibility, or automatic gating. The algorithm used in the latter case is proprietary and can produce unsatisfactory results, especially when applied to ddPCR data obtained from formalin-fixed paraffin-embedded (FFPE) samples, as exemplified in Figure 5A.

Two third-party tools for automatic gating of ddPCR data have been described to date: ‘definetherain’ by Jones *et al.* ^3^ and ’ddpcRquant’ by Trypsteen *et al.* ^4^. However, both are limited to single-channel ddPCR data and are therefore not applicable to increasingly common two-channel experiments such as shown in Figure 1. Given the lack of tools for such analyses, users must currently resort to manual droplet gating.

## Methods

### Overview

To improve automated droplet assignments as well as permit visualization of ddPCR datasets, we have developed *ddpcr*, an R package that can be used to explore, visualize, and analyze two-channel ddPCR data. The R language ^5^ was chosen because it is open-source and cross-platform, which allows anyone to use it freely on any operating system. R is also a popular language in the field of computational biology, and is the main data analysis language for many scientists. To improve access and ease of use, we also implemented an interactive web application using Shiny ^6^, through which one can run the analysis using a simple point-and-click interface.

*ddpcr* has been thoroughly tested using R versions 3.2.3 and 3.3.0 on both Windows 7 and Ubuntu 14.04.2 machines. However, the package is likely to run on any machine with a working installation of R.

### Plate object

The most important object in the *ddpcr* package is the ddpcr_plate object, or simply referred to as the "plate object". A plate object represents all the data for experiments conducted on a 96-well PCR plate. It gets created either by loading ddPCR input data files (see ‘Data import’) into a new plate object, or by loading an existing plate object that was previously saved to disk. A plate object contains all the information required to analyze the droplets within each well of a particular ddPCR plate. A plate object is both the input and output of all the core analysis functions.

### Workflow

To use the *ddpcr* package, it must first be installed and loaded.



                        install.packages("ddpcr")
library("ddpcr")
                    


**Figure 2.**
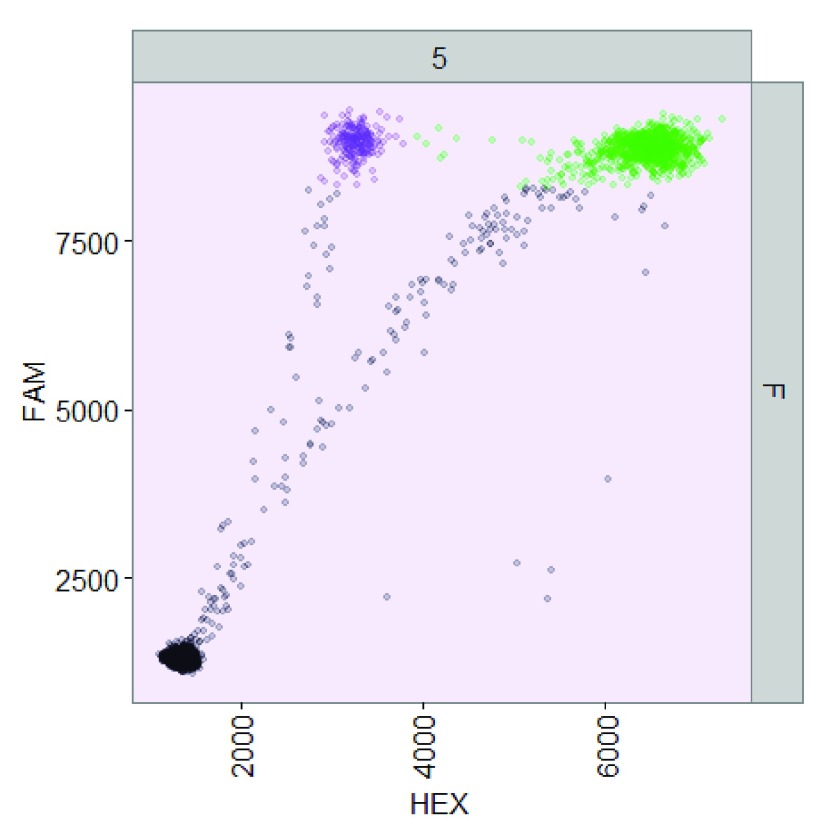
ddPCR data from well F05 of the sample dataset analyzed using *ddpcr*.

A very simple analysis workflow using a sample dataset can be performed using the following code, with the result of the code shown in Figure 2:



                        dir <– sample_data_dir()
my_data <– new_plate(dir, type = plate_types$fam_positive_pnpp)
my_data <– subset(my_data, "F05")
my_data <– analyze(my_data)
plot(my_data, show_drops_empty = TRUE, show_grid_labels = TRUE)
                    


**Figure 3.**
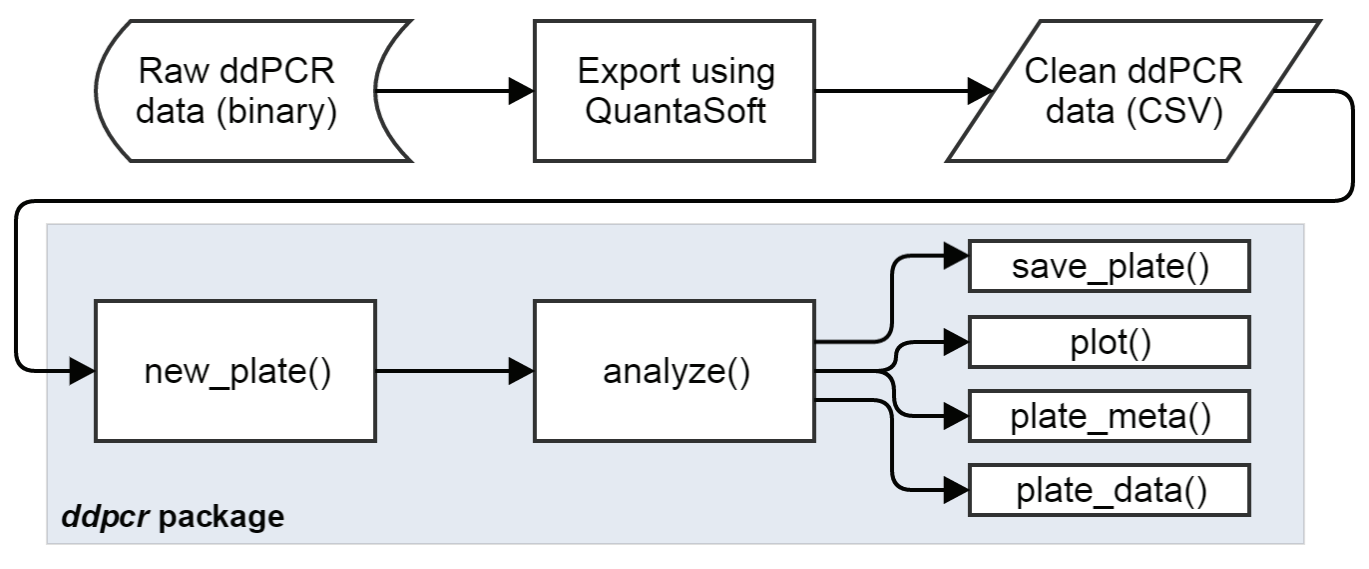
Basic workflow for analyzing ddPCR data using the *ddpcr* package.

While *ddpcr* contains dozens of functions, most analyses will follow a similar pattern: load ddPCR data into R using the new_plate() function, run the automated analysis using analyze(), and then explore the results using a variety of functions ( Figure 3). The plot() function is used to visualize a dataset using *ggplot2* ^7^, while the plate_meta() and plate_data() functions return the dataset’s metadata and droplet grouping data as R data frames, respectively. The save_plate() function can be called at any time to save the current state of the dataset to disk in a format that can be loaded back into *ddpcr*.

The example code above uses a sample dataset, but in order to use new data, ddPCR data must be exported from QuantaSoft, as described in the next section. For more complex analysis or customizing the analysis parameters, see the full list of functions available by running ?ddpcr.

### Data import

Before beginning analysis on a novel dataset, the first step is to import the ddPCR droplet fluorescence data into R. The raw data obtained from the fluorescence detector is encoded in a proprietary format that cannot be read by any software other than QuantaSoft, so the data must first be opened in QuantaSoft and exported into an accessible file format. QuantaSoft offers an option to export the droplet event data as a set of CSV (comma-separated values) files, as well as an option to export a metadata file that contains information on each well ( Supplementary Figure 1 and Supplementary Figure 2). These CSV files are used as the input to *ddpcr*.

### Analysis algorithm

The analysis automatically gates droplets into unique clusters using kernel density estimation and Gaussian mixture models applied to the droplet fluorescence amplitudes. The full algorithm is explained in detail in a package vignette. The main analysis steps are:

- Identify and exclude wells with a failed ddPCR reaction.
- Identify and exclude outlier droplets, defined as those exhibiting a set of fluorescence amplitude signals characteristic of an error in the fluorescence readout.
- Identify and exclude empty droplets — those displaying a set of signals indicative of complete absence of DNA template.
- Calculate the starting concentration of each template in the sample, defined as the number of copies per microlitre of input.
- Assign droplets into clusters by gating the droplets based on their fluorescence amplitudes. QuantaSoft’s automatic gating does not account for rain droplets and therefore can produce inaccurate results when the density of rain falls above a threshold. The gating algorithm in *ddpcr* accounts for rain and is therefore better able to distinguish clusters in clinical samples, such as FFPE samples, for which significant rain is often observed. Manual gating is also available in *ddpcr* to permit secondary verification of results.
- Count the number of droplets in each cluster.

### Implementation

***Plate objects are lists.*** Every S3 object in R has a base type upon which it is built. The plate object is implemented as an S3 object of class ddpcr_plate with the R list as its base type. Using a list allows for an easy way to bundle together the several different R objects describing a plate into one. All information required to analyze a plate is part of the plate object. Every plate object contains a set of nine elements that together fully describe and reproduce the current state of the dataset: plate_data, plate_meta, name, params, status, clusters, steps, dirty, version.

***Using S3 to override base generic functions.*** Since the plate object is an S3 object, it can benefit from the use of generic functions. There are three common generic functions that the plate object implements: print(), plot(), and subset(). The print() method does not take any extra arguments and is used to print a summary of a plate object in a visually appealing way to the console. It gives an overview of the most important parameters of the plate such as its name and size. The plot() method generates a scatter plot of every well in the dataset and can be highly customizable using the many arguments it supports. While the base plot() method in R uses base R graphics, the plot() method for ddpcr_plate objects uses the *ggplot2* package ^7^. The subset() generic is overridden by a method that is used to retain only a subset of wells from a larger plate.

***Plate types.*** A ddPCR assay can be characterized by the droplet populations that are expected to arise after amplification. For example, in a (FAM ^+^)/(FAM ^+^HEX ^+^) assay (such as Figure 1) it is expected that most of the non-empty droplets will either be FAM ^+^HEX ^+^ or FAM ^+^, but not HEX ^+^. Similarly, a (HEX ^+^)/(FAM ^+^HEX ^+^) assay means that there are expected to be no droplets that are only FAM+. To describe these two types of assays, we define the term "PN/PP" (positive-negative/positive-positive). This name is a reflection of the expected populations of non-empty droplets: one population of singly-positive droplets (such as HEX ^+^ or FAM ^+^), and one population of double-positive droplets.

This characterization of a ddPCR experiment defines the plate type of a plate object, and it determines what type of analysis to run on the data. The default and most basic plate type is ddpcr_plate, which can be used for any ddPCR dataset. Running the analysis on a plate of this type will perform the first few analysis steps of identifying failed wells, outlier droplets, and empty droplets, but will not carry out the automated gating. Since in PN/PP-type experiments there is a rough expectation of where the droplets should be, automated gating can ensue on plates of that type.

***Using S3 to support inheritance*** Inheritance means that every plate type has a parent plate type from which it inherits all its features, while specific behaviour can be added or modified. In *ddpcr*, transitive inheritance is implemented, which means that features are inherited from all ancestors rather than only the most immediate one. Multiple inheritance is not supported, meaning that each plate object can only have one parent.

The notion of inheritance is an important part of the *ddpcr* package, as it allows ddPCR data from different assay types to share many properties. For example, PN/PP assays are first treated using the analysis steps common to all ddPCR experiments, and then gated with an assay-specific step, so PN/PP assays can be thought of as inheriting the analysis from general ddPCR assays. Furthermore, the two types of PN/PP assays share many similarities, so they both inherit from a common PNPP plate type. Another benefit of inheritance in *ddpcr* is that it allows users to easily extend the functionality of the package by adding custom ddPCR plate types to gate different types of experiments. More information, including a fully worked example, on how to add a new plate type can be found in the package vignette (see ‘Software availability’).

### Shiny web application

The *ddpcr* package includes a web application that allows users to perform an analysis of ddPCR data in an interactive visual environment. The web application, written using the Shiny package v0.11 ^6^, implements most of the features available in the *ddpcr* package and makes them accessible via a simple point-and-click interface. The Shiny application can be a useful tool for persons not comfortable with R programming or simply as a more convenient way to perform an analysis. However, since the web application only supports a curated subset of the *ddpcr* functions, it is not as powerful as using the command-line interface.

**Figure 4.**
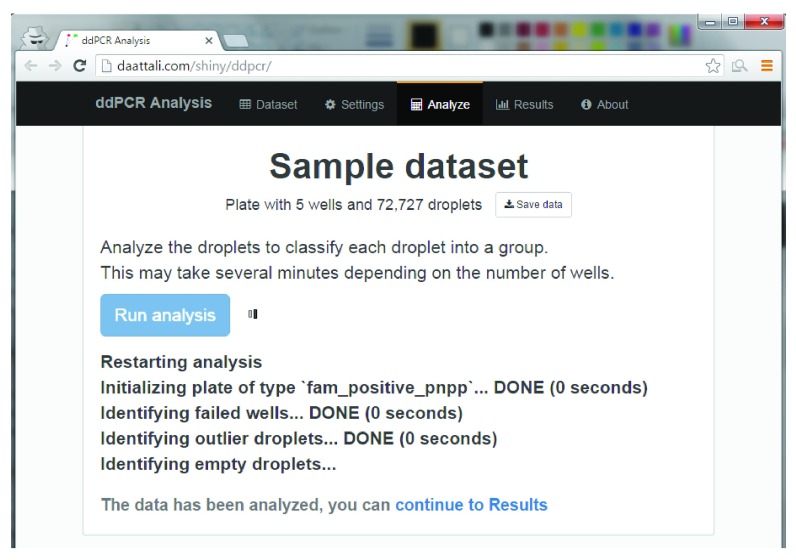
Screenshot from the *ddpcr* web application during an analysis of the sample ddPCR dataset.

The *ddpcr* Shiny application includes four main tabs that mimic the natural flow of a ddPCR analysis ( Figure 4): upload a dataset, configure analysis parameters, analyze the plate, and explore the results. At any point during the session, the current plate object can be downloaded and saved, and can be loaded into either the R command-line or the web application at a later time to continue the analysis.

The application is freely available online at http://daattali.com/shiny/ddpcr and is hosted on a server located in San Francisco, California. All data that is uploaded to the application is deleted when a user session ends, and none of the data is stored permanently. However, some users may prefer to run the application locally, which can be done using the ddpcr::launch() function.

## Use case

Raw ddPCR data from application of the ddPCR assay against BRAF-V600 mutations This data can be loaded and displayed in QuantaSoft™. Column 12 on the plate is from a different experiment and is not considered part of the dataset. Click here for additional data file. Copyright: © 2016 Attali D et al. 2016 Data associated with the article are available under the terms of the Creative Commons Zero "No rights reserved" data waiver (CC0 1.0 Public domain dedication).

The set of exported CSV files of the data presented in Dataset 1 Click here for additional data file. Copyright: © 2016 Attali D et al. 2016 Data associated with the article are available under the terms of the Creative Commons Zero "No rights reserved" data waiver (CC0 1.0 Public domain dedication).

We have applied *ddpcr* to data ( Dataset 1) from a novel ddPCR assay against somatic point mutations in the *BRAF*-V600 codon that was applied to FFPE specimens from a cohort of colorectal cancer (CRC) patients ^8^. V600 mutations are observed in approximately 10% of colorectal tumours ^9^ and their detection in CRC patients helps determine disease prognosis and treatment regimen. Through its droplet gating algorithm, *ddpcr* accurately identified droplet clusters and the number of droplets within each to provide the information needed to compute the frequency of mutated *BRAF* genes ( Supplementary Figure 3).

To assess the accuracy of results from *ddpcr*, we compared *BRAF*-V600 mutation frequencies determined from the output of *ddpcr* with results obtained by two independent methods. V600 mutation frequencies computed from automated *ddpcr* results were within 3% of those obtained by manual analysis of the ddPCR data by an experienced operator ( Supplementary Figure 4 and Supplementary Table 1). In addition, the *BRAF*-V600 status for each sample in the entire cohort was classified as mutant or wild-type by a certified pathologist using an immunohistochemical staining assay ^8^. We obtained complete agreement between the pathologist’s binary classification of *BRAF* status and that determined using *ddpcr*.

**Figure 5.**
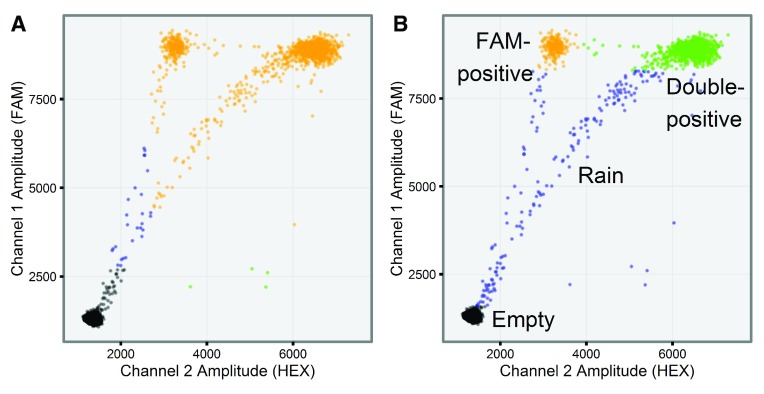
Comparison between droplet gating in ( **A**) QuantaSoft and ( **B**) *ddpcr*. Both tools analyzed the same ddPCR experiment (well F05) from an assay designed to quantify wild-type (double-positive) and mutant (FAM-positive) alleles of the *BRAF* gene. ( **A**) QuantaSoft failed to assign the double-positive and FAM-positive droplets into unique clusters, instead assigning all droplets recording a high FAM signal to a single cluster; ( **B**) *ddpcr* assigned droplets into one of three uniquely identified clusters (double-positive (green), FAM-positive (orange), and empty (black)), or rain (blue).

We also analyzed the same dataset using QuantaSoft version 1.6.6. FAM-positive and double-positive droplets were not recognized as distinct clusters in 9 out of the 16 mutant-positive *BRAF* samples ( Figure 5A).

## Discussion

We present *ddpcr*, an R package that allows users to analyze ddPCR data and explore the results, both programmatically using R and via an interactive web application. To demonstrate clinical utility, a case study performed on a cohort of CRC patients showed that *BRAF*-V600 mutation frequencies determined using *ddpcr* are verified using two independent methods. The analysis runtime was 17 seconds, observed on a 64-bit Ubuntu 14.04.2 machine with 512MB of RAM and a single core Intel(R) Xeon(R) CPU E5-2630 at 2.30GHz. The package documentation includes details on extending the package, explanations of the algorithms used, and a walkthrough of a fully worked example.

## Data availability

The data referenced by this article are under copyright with the following copyright statement: Copyright: © 2016 Attali D et al.

Data associated with the article are available under the terms of the Creative Commons Zero "No rights reserved" data waiver (CC0 1.0 Public domain dedication). 

F1000Research: Dataset 1. Raw ddPCR data from application of the ddPCR assay against *BRAF*-V600 mutations, 10.5256/f1000research.9022.d126032 ^10^

F1000Research: Dataset 2. The set of exported CSV files of the data presented in Dataset 1., 10.5256/f1000research.9022.d126033 ^11^

Dataset 1 is also available as a sample dataset within the *ddpcr* package. To access the data via the web application, select the tab *Use sample dataset*, choose *Large dataset*, and then click *Load data*. To access the data in R, run the following command to store the dataset as a plate object: my_data <- ddpcr::sample_plate("large").

## Software availability

Software available from: http://cran.r-project.org/package=ddpcr or https://github.com/daattali/ddpcr

The free web tool can be accessed online at: ( http://daattali.com/shiny/ddpcr); or run locally via the *ddpcr* package with the command ddpcr::launch().

Latest source code: https://github.com/daattali/ddpcr

Archived source code at time of publication: https://dx.doi.org/10.6084/m9.figshare.3423725 ^12^

License: MIT

## Supplementary material

**Figure 1.** Exporting droplet fluorescence data from QuantaSoft.

**Figure 2.** Exporting plate metadata from QuantaSoft.

**Figure 3.** Automated *ddpcr* droplet gating results for raw assay output for the cohort of 32 CRC patient samples. The numbers show the calculated *BRAF*-V600 mutation frequency, defined as the ratio of FAM-positive droplets to the sum of FAM-positive and double-positive droplets. Background colours: green = sample is classified as wild-type, purple = sample is classified as mutant, grey = failed ddPCR run.

**Figure 4.** Comparison of the mutation frequency in each patient sample as calculated automatically by *ddpcr* vs that determined independently manually by an expert technician. The grey line represents the *y* = *x* line.

**Table 1.** Comparison of the mutation frequency in each patient sample as calculated automatically by *ddpcr* vs that determined independently manually by an expert technician.
