# Supplementary figures and images for: ddpcr: an R package and web application for analysis of droplet digital PCR data
Source: F1000Res. 2016 Jun 17;5:1411. [Version 1] doi: 10.12688/f1000research.9022.1 (PMC5031129; doi:10.12688/f1000research.9022.1)

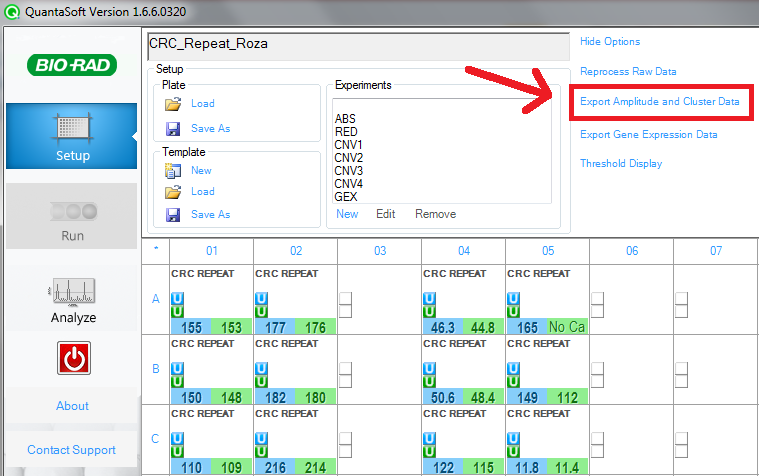

Supplement: Supplementary file 3 [file f1000research-5-9706-s0002.tgz › 02e5a0e9-4690-405e-961c-c9f85de5f918.png]

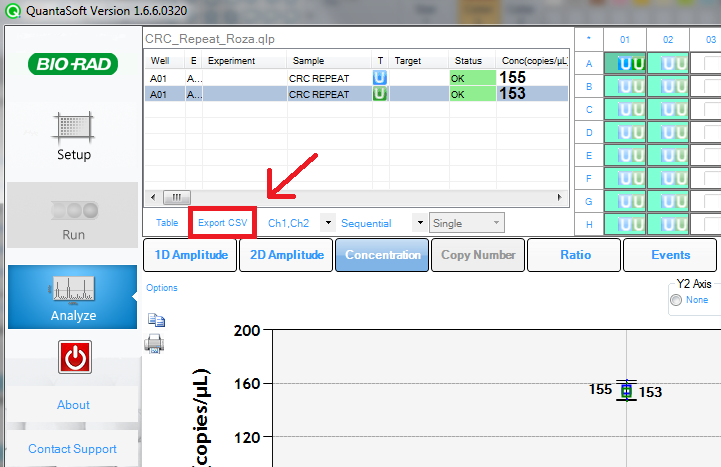

Supplement: Supplementary file 4 [file f1000research-5-9706-s0003.tgz › 8985e156-188b-4009-b612-d63e98e82510.png]

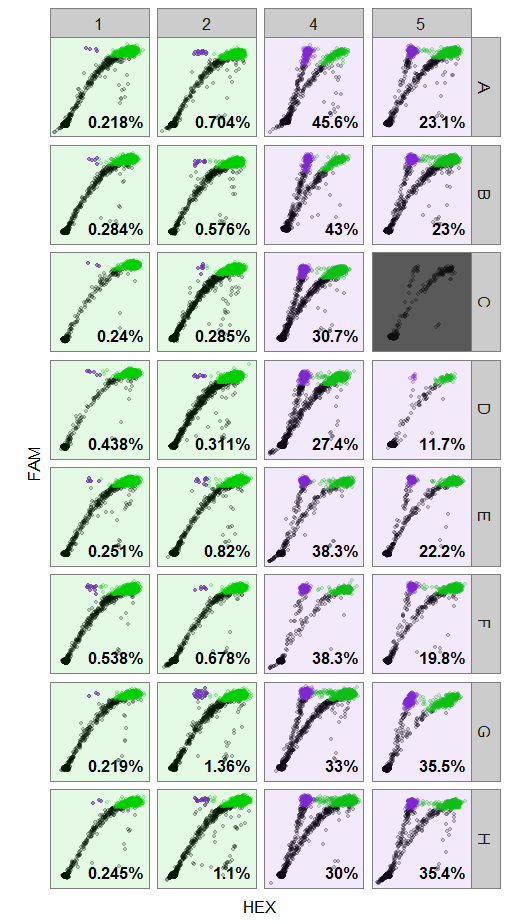

Supplement: Supplementary file 5 [file f1000research-5-9706-s0004.tgz › 34e1ff63-bef5-41cf-8b17-257b392b220c.tif]
