# Supplementary material for: ddpcr: an R package and web application for analysis of droplet digital PCR data
Source: F1000Res. 2016 Jun 17;5:1411. [Version 1] doi: 10.12688/f1000research.9022.1 (PMC5031129; doi:10.12688/f1000research.9022.1)
Supplement: Peer review history

# Peer review: Referee response for version 1

**DOI:** 10.5256/f1000research.9706.r15716

**Published:** 2016-09-14

**Authors:** Stephanie L. Hazlitt, Andy Teucher

**Affiliations:** 1 Ministry of Environment - Province of British Columbia, Victoria, BC, Canada

The software tool article 'ddpcr: an R package and web application for analysis of droplet digital PCR data' is well-written and includes sufficient detail for the reader to assess the tool's construction, implementation and outputs. The ddpcr R package (v1.5) functions well and includes clearly-written, detailed vignettes to support the user and understand what is happening 'under the hood'. In addition, the source code is openly available on GitHub, which allows advanced R users to investigate details of the implementation. In general, we recommended more explicit links in the paper to supporting vignettes and the package README.

 In addition to ddpcr being a useful tool for standard data science steps (get data, visualize data, analyze data), it also provides the ddPCR community with a well-documented, new analytical methodology for ddpcr clustering and hence opportunities for assessing robustness around clustering techniques in general in this field. This contribution could be made more clear in the Introduction and/or Motivation sections.

 Figure 5--referenced multiple times in the Introduction--could be moved alongside the text for the reader.

 The Methods structure could be arranged to follow standard data science steps and Figure 3: (1) get data [raw, export, csv] (2) import data [new_plate()] (3)analyze data [analyze()]. There is no section on (4) visualize data [plot()], and the likely user cycle of analyze-visualize-change analysis parameters-analyze-visualize. With clustering, I am guessing this will be a common and important set of repeat steps. I would end the section with the example workflow code to reinforce the steps.

 The Analysis sub-section might benefit from some supporting references for the selected analyses (e.g. kernel density estimation, Gaussian mixture models), url links to the ddpcr::analysis vignette in GitHub, and if the paper word-limit allowed, a bit more detail in the paper itself, especially for the 'assign droplets into clusters' step.

 The examples show rain being identified in the FAM (empty vs filled; vertical direction), but not between FAM+ and FAM+HEX+ (mutant vs wildtype; horizontal direction). Is this because the algorithm doesn't allow for this, or because all of the droplets in that range could be classified as FAM+ or FAM+HEX+?

 Also in the Analysis sub-section, a description of the next_step() function would be useful for those who want to understand each step in the process. A potential enhancement to the package would be a plot function/method for each step that visually displays the results of each step in the analysis.

 It might be useful to synchronize the language used to describe the clusters with the attribute language in output objects in the package. For example, in the paper and vignettes clusters are "double positive, FAM+, HEX+, or double negative". In clusters() the clusters are POSITIVE NEGATIVE RAIN EMPTY etc. The plate_data function outputs clusters as numbers 1 through 7. The plate_meta function outputs clusters as mutant_num and wildtype_num. With a bit of sleuthing it is clear you can define the resulting cluster names with new_plate(), which is useful but may not be intuitive for many users.

 Other minor suggestions for potential enhancements after a test drive of ddpcr:

 - Plotting:

       - an addition of a Legend for the plot() would be a very useful

       - show_grid_labels = TRUE as default?

       - visually distinguish rain from empty? Figure 5 in the paper shows this, but the plot   

         generated by running the code in the 'Workflow' section does not.

 - The plate_meta data.frame in a ddpcr_plate object could be a tibble for better printing

 - The plate_meta data.frame (after analysis) could also display the rain count

We have read this submission. We believe that we have an appropriate level of expertise to confirm that it is of an acceptable scientific standard.

# Peer review: Referee response for version 1

**DOI:** 10.5256/f1000research.9706.r14448

**Published:** 2016-07-04

**Authors:** Timothy J. Triche Jr

**Affiliations:** 1 Jane Anne Nohl Division of Hematology, USC/Norris Comprehensive Cancer Center, Keck School of Medicine of USC , Los Angeles, CA, USA

The ddpcr software is extensively documented and works as described. There are some minor changes that might be relevant (e.g. in ddpcr v1.4, the plot contains a percentage estimate in the lower right hand quadrant which is not shown in figure 1, and the X- and Y-axes are labelled slightly differently in the actual output from running the example. Overall, however, the package offers both a powerful tool for end users which advances the state of the art, and also a foundation for further algorithmic development. Cross-pollination between the flow cytometry community and ddpcr in the R ecosystem will likely lead to advances that would not otherwise have occurred. 

 Figure 3 should be moved alongside the text in the paragraph "Analysis algorithm". 

 Figure 5 is a powerful demonstration of the rationale for the library's creation and could perhaps stand to be moved up in the body of the paper. Standard practice is to provide a reason for the user to care about a method (e.g. figure 5), then describe the nuts and bolts of the implementation. There is a reason that this is standard practice. 

 Time permitting, this reviewer has sought and obtained some much more challenging samples. In a revised or separate publication, it would be of interest to compare the software's performance on these ddPCR runs (where 1 in 10000 cells carries a mutation, but that 1 in 10000 is verified by multiple orthogonal sequencing methods) and determine whether the algorithmic flexibility of the ddpcr package would allow users to automate what is currently a highly technical process for detecting rare mutations. Unfortunately, the manufacturer has been unhelpful in converting this data to a usable format within the timeline of the review, so I cannot in good conscience delay it further.

 Outside of the scope of this paper, but relevant to the introduction, reproducibility and comparability would be much improved if the raw .QLP files could be parsed by R itself, making exchange and sharing of data far easier.  Inquiries placed to Bio-Rad regarding the file format and the QuantaSoft package went unanswered, suggesting that (as with Illumina and their .IDAT format) the eventual solution will be to reverse engineer the format and brute-force the problem. It is unfortunate that some scientific instrument vendors appear to value imaginary profits over actual scientific merit, but such is life. Reverse engineering the file format is not a reasonable request here, but represents a future direction to further improve reproducibility of this important analytical technique. Existing tools rely upon ddpcr data provided in .CSV format; the first to elide this requirement (and that of a $5000 software package merely to review output) will be a notable step towards transparency for an increasingly powerful genotyping technique.

 In conclusion, the ddpcr implementation and its extensive documentation (here in this paper and in the copious examples provided with the package) represent a solid foundation for further methodological improvements to an important assay platform.

I have read this submission. I believe that I have an appropriate level of expertise to confirm that it is of an acceptable scientific standard.

# Response

**Published:** 2016-08-19

**Authors:** Dean Attali

Thank you for the thorough and prompt review. I will address the comments once I get a review from the second reviewer (it's taking longer than expected to find someone!)
